# Assessing the efficacy of 2D and 3D CNN algorithms in OCT-based glaucoma detection

**DOI:** 10.1038/s41598-024-62411-6

**Published:** 2024-05-23

**Authors:** Rafiul Karim Rasel, Fengze Wu, Marion Chiariglione, Stacey S. Choi, Nathan Doble, Xiaoyi Raymond Gao

**Affiliations:** 1 Department of Ophthalmology and Visual Sciences, The Ohio State University, Columbus, OH 43212 USA; 2 Department of Biomedical Informatics, The Ohio State University, Columbus, OH 43210 USA; 3 Division of Human Genetics, The Ohio State University, Columbus, OH 43210 USA; 4 College of Optometry, The Ohio State University, Columbus, OH 43210 USA

**Keywords:** Optic nerve diseases, Computational science

## Abstract

Glaucoma is a progressive neurodegenerative disease characterized by the gradual degeneration of retinal ganglion cells, leading to irreversible blindness worldwide. Therefore, timely and accurate diagnosis of glaucoma is crucial, enabling early intervention and facilitating effective disease management to mitigate further vision deterioration. The advent of optical coherence tomography (OCT) has marked a transformative era in ophthalmology, offering detailed visualization of the macula and optic nerve head (ONH) regions. In recent years, both 2D and 3D convolutional neural network (CNN) algorithms have been applied to OCT image analysis. While 2D CNNs rely on post-prediction aggregation of all B-scans within OCT volumes, 3D CNNs allow for direct glaucoma prediction from the OCT data. However, in the absence of extensively pre-trained 3D models, the comparative efficacy of 2D and 3D-CNN algorithms in detecting glaucoma from volumetric OCT images remains unclear. Therefore, this study explores the efficacy of glaucoma detection through volumetric OCT images using select state-of-the-art (SOTA) 2D-CNN models, 3D adaptations of these 2D-CNN models with specific weight transfer techniques, and a custom 5-layer 3D-CNN-Encoder algorithm. The performance across two distinct datasets is evaluated, each focusing on the macula and the ONH, to provide a comprehensive understanding of the models’ capabilities in identifying glaucoma. Our findings demonstrate that the 2D-CNN algorithm consistently provided robust results compared to their 3D counterparts tested in this study for glaucoma detection, achieving AUC values of 0.960 and 0.943 for the macular and ONH OCT test images, respectively. Given the scarcity of pre-trained 3D models trained on extensive datasets, this comparative analysis underscores the overall utility of 2D and 3D-CNN algorithms in advancing glaucoma diagnostic systems in ophthalmology and highlights the potential of 2D algorithms for volumetric OCT image-based glaucoma detection.

## Introduction

Glaucoma, a neurodegenerative disease affecting nearly 80 million people globally^1^, is characterized by the progressive degeneration of retinal ganglion cells, leading to vision impairment^1–5^. It stands as a leading contributor to irreversible vision loss, often going undetected until considerable damage has occurred^6–8^. In developed countries, almost half of the individuals with glaucoma remain undetected^9,10^, while in developing countries, the number of undetected cases can reach as high as 90%^11^. Therefore, early, and accurate diagnosis is essential to reduce further visual deterioration. In this regard, advances in optical coherence tomography (OCT) present a promising avenue, offering a viable means for early glaucoma detection.

By providing detailed visualization of crucial ocular structures, such as the macula and optic nerve head (ONH)^12–14^, OCT offers an exceptional opportunity to study the effects of vision-related diseases such as glaucoma. While automated algorithms have significantly streamlined the analysis of intricate details in OCT, the interpretation of data from these automated systems still requires expert input for accurate predictions. Consequently, deep learning (DL) based algorithms have garnered considerable popularity in streamlining OCT image analysis, providing substantial insights for deriving meaningful conclusions^15–19^.

Previous reports on DL-based algorithms for predicting glaucoma from OCT images showed a predominant use of the 2D convolutional neural network (CNN) compared to 3D-CNN models for glaucoma detection. For instance, Mehta et al.^15^ proposed a multimodal model leveraging various ocular and clinical data for glaucoma detection. Their OCT-based model employs a 2D-CNN-based DL framework with a Densenet201^15^ backbone to train on each B-scan from OCT volumes obtained from the UK Biobank (UKB) Supplementary Materials. The model provides final glaucoma predictions by collectively analyzing each B-scan of the OCT volume, achieving a notable AUC score of 0.950.

Additionally, Christopher et al.^20^ introduced a 2D-CNN-based approach that capitalizes on the wide-angle swept-source OCT images to extract relevant RNFL features for improved glaucoma classification. Their method has been shown to surpass glaucoma detection based on conventional metrics, such as circumpapillary RNFL thickness, standard automated perimetry, and frequency-doubling technology visual field tests. Furthermore, Garcia et al.^21^ introduced an innovative algorithm that combines manually crafted features with a 2D-CNN algorithm, incorporating tailored residual and attention modules to achieve robust glaucoma detection. This combination enhances the discrimination between healthy, early, and advanced glaucoma samples, resulting in superior classification accuracy.

In contrast to the 2D-CNN-based methodology, Maetschke et al.^22^ proposed a 3D-CNN algorithm for glaucoma detection. Their 3D-CNN-based model directly classifies healthy and glaucomatous eyes from unsegmented OCT volumes of the ONH region, attaining a robust AUC of 0.940 on the test dataset. Following a similar trajectory, Yasmeen et al.^23^ introduced an attention-guided 3D DL model for analyzing OCT images to detect glaucoma. The proposed model operates through three pathways with different inputs but the same model architecture and, when validated, achieved an AUC of 0.983, outperforming traditional models and other machine learning methods. Despite the promising outcomes of 2D and 3D-CNN algorithms in glaucoma detection, our review highlights a research gap. Specifically, research examining the 2D and 3D-CNN algorithms for glaucoma detection, particularly through weight transfer techniques to enhance 3D models’ performance across diverse OCT datasets, is notably limited.

Considering that macular and ONH OCT inherently offers volumetric imaging data and given the scarcity of pre-trained 3D models trained on extensive datasets, the importance of examining the strengths of current 2D and 3D-CNN models in detecting glaucoma from OCT volumes cannot be underestimated. While 2D-CNN models may not utilize volumetric information available in OCT volumes, they benefit from a wealth of pre-trained models, offering better generalization in smaller datasets. In contrast, although 3D-CNN models can leverage the volumetric information, they are often limited by a notable scarcity of pre-trained models and large volume datasets. Even though there are ways to incorporate transfer learning, i.e., through dimensionality expansion of 2D-CNN models^24–26^, such techniques remain approximate and possibly not fully optimized.

Therefore, our study investigates the effectiveness of select 2D and 3D-CNN algorithms for OCT-based glaucoma detection by leveraging DL models, including 2D and 3D versions of ResNet18^27,28^ and DenseNet121^29^, and a 5-layer 3D-CNN-Encoder model^22^. In this study, we utilized dimensionality expansion to adapt 2D models for 3D applications, enabling weight transfer from an ImageNet1k-trained 2D model to improve a 3D model’s performance. Through extensive evaluations using two publicly available volumetric OCT image datasets, the first available through the UKB^30^ focusing on the macula and the other published by Maetschke et al.^22^ focusing on ONH—this study underscores the robust performance of 2D-CNN algorithms in detecting glaucoma compared to their 3D counterparts and the weight transfer method tested in this study. These findings underscore the strengths of 2D-CNN in glaucoma detection via volumetric OCT images and point out the limitations of current weight transfer techniques for 3D-CNN models. They also highlight the potential of 3D algorithms and the necessity for future research to improve 3D-CNNs’ performance in glaucoma detection. Therefore, the findings of this study have the potential to transform diagnostic approaches in the field of ophthalmology using OCT-based datasets.

## Results

**Figure 1:**
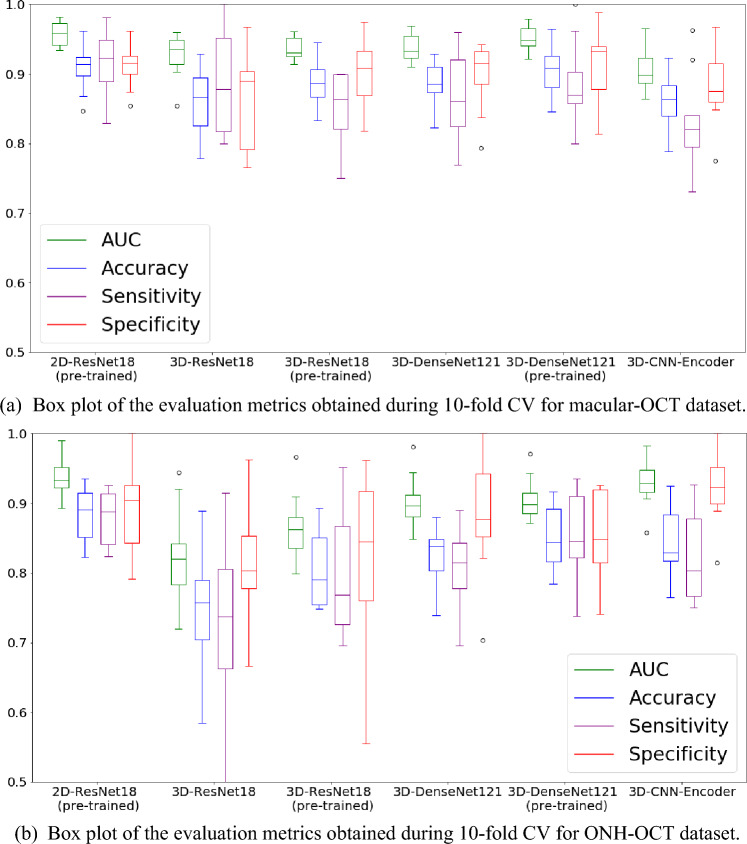
Box plots of evaluation metrics AUC, accuracy, sensitivity, and specificity obtained through tenfold cross-validation on the test data. This figure presents the box plot of the evaluation metrics AUC, accuracy, sensitivity, and specificity obtained through tenfold cross-validation on the test data. Subfigure (**a**) illustrates the results for the Macular-OCT model, while subfigure (**b**) displays the results for the ONH-OCT model. The AUC values box plot reveals that, for both macular-OCT and ONH-OCT datasets, the 2D CNN model delivers superior overall results. Additionally, the 2D model yields robust results for other essential metrics such as accuracy and sensitivity. Among the 3D CNN models, the pre-trained 3D-ResNet18 outperformed the 3D-ResNet18 model trained from scratch. Notably, the 3D-CNN-Encoder exhibits superior performance compared to the 3D-ResNet18 models when tested on the ONH-OCT dataset, while delivering subpar performance on the macular-OCT dataset. *AUC* area under the receiver operating characteristic curve, OCT optical coherence tomography, *macular-OCT* macural-OCT, *ONH-OCT* optic nerve head OCT.

Table 1 presents the comparative performance of various models trained on the macular-OCT and ONH-OCT test datasets. The evaluation metrics include tenfold cross-validation (CV) average values for the AUC, accuracy, sensitivity, and specificity. Accuracy, sensitivity, and specificity scores, calculated based on the Youden Index, are specific to datasets and models, thus providing a qualitative assessment of the model’s performance. Meanwhile, Fig. 1 displays the box plots of the evaluation metrics obtained from the macular-OCT and ONH-OCT test datasets during the tenfold CV study.

**Table 1:** Comparative performance of models on macular-OCT and ONH-OCT test datasets: average AUC, accuracy, sensitivity, and specificity values from tenfold CV.

Models	Pre-trained	macular-OCT				ONH-OCT			
		AUC	ACC	Sen	Spe	AUC	ACC	Sen	Spe
2D-ResNet18	Yes	**0.960**	0.901	**0.891**	0.913	**0.943**	**0.890**	**0.917**	0.910
3D-ResNet18	No	0.928	0.863	0.885	0.861	0.823	0.750	0.730	0.820
3D-ResNet18	Yes	0.937	0.890	0.850	0.900	0.863	0.810	0.810	0.820
3D-DenseNet121	No	0.938	0.901	0.848	**0.923**	0.889	0.818	0.806	0.857
3D-DenseNet121	Yes	0.945	**0.905**	0.878	0.915	0.906	0.840	0.829	0.880
3D-CNN-Encoder	No	0.910	0.852	0.820	0.873	0.931	0.844	0.821	**0.921**

*CV* cross-validation, *CNN* convolutional neural network, *OCT* optical coherence tomography, *ONH-OCT* optic nerve head OCT, *Acc* accuracy, *Sen* sensitivity, *Spe* specificity.

For the macular-OCT dataset, the pre-trained 2D-ResNet18 model emerged as the top performer, achieving an AUC of 0.960, and showcasing an accuracy, sensitivity, and specificity of 0.910, 0.891, and 0.913, respectively. These results were closely followed by the pre-trained 3D-ResNet18 and 3D-DenseNet121, which posted an AUC of 0.937 and 0.945, respectively. Conversely, the 3D-ResNet18, 3D-DenseNet121, and 3D-CNN-Encoder models, trained from scratch on this dataset, recorded AUCs of 0.928, 0.938, and 0.910, respectively, highlighting the advantage of utilizing weight transfer technique to improve 3D models.

Regarding the ONH-OCT dataset, the 2D ResNet18 model once again surpassed its 3D counterparts tested in this study, achieving an AUC of 0.943, an accuracy of 0.890, a sensitivity of 0.917, and a specificity of 0.803. Among the 3D models utilized for this dataset, the 3D-CNN-Encoder model outperformed the other 3D models by achieving an AUC of 0.931. Meanwhile, the 3D-ResNet18 and its pre-trained version achieved AUCs of 0.823 and 0.863, respectively, while the 3D-DenseNet121 and its pre-trained counterpart recorded AUCs of 0.889 and 0.906, respectively. The diminished AUCs observed in 3D-ResNet18 and 3D-DenseNet121 models on the ONH-OCT dataset might potentially be attributed to a predisposition for overfitting. We suspect that this overfitting is due to the inherent complexity of the 3D models employed, compounded by the smaller size of the dataset and lower resolution of the images utilized in this study.

Moreover, the box plot analysis (as depicted in Fig. 1) of the AUCs obtained from the tenfold CV emphasizes the steadfast performance of the 2D model. This illustration also demonstrates an overall strong performance achieved by the pre-trained 3D CNN models compared to their counterparts trained from scratch. Therefore, a significantly larger dataset could greatly enhance the 3D model’s performance. Overall, as indicated by the results presented in Table 1 and Fig. 1 for the models tested in this study, the 2D-ResNet18 model consistently exhibited higher performance across both volumetric OCT datasets in the tenfold CV.

## Discussion

Glaucoma, a prevalent neurodegenerative disease affecting millions worldwide, necessitates early and precise diagnosis to mitigate irreversible vision loss. OCT has emerged as a powerful tool in ophthalmology, offering detailed insights into ocular structures. In this study, we investigated the effectiveness of select 2D and 3D CNN algorithms for glaucoma detection using both macular and ONH OCT images.

Our findings demonstrate that the pre-trained 2D-ResNet18 model provides robust results when applied to volumetric OCT datasets. The consistent performance of the 2D-ResNet18 model on both macular-OCT and ONH-OCT datasets indicates its potential to improve diagnostic practices in ophthalmology, especially considering the scarcity of large glaucoma datasets or the lack of 3D-CNN models trained on extensive datasets. Notably, compared to the 3D-CNN models tested in this study, the 2D-ResNet18 model achieved the highest AUC scores of 0.960 and 0.943 on macular-OCT and ONH-OCT, respectively.

Furthermore, when considering the comparison between 2D and 3D CNN algorithms, it is beneficial to highlight several advantages of 2D-CNN models. 2D-CNN models encompass a wider array of available pre-trained models, decreased computational complexity in model training, easier to interpret and visualize intermediate model layers due to lower dimensionality, simplified data augmentation due to existing libraries and lower dimensionality, and enhanced scalability. Moreover, in light of data scarcity and appropriate pre-trained 3D models, the application of 2D-CNNs is not only limited to glaucoma detection, it can be extended to other medical fields with reasonable accuracy and performance^31–33^. Consequently, considering the results presented and the evident benefits of 2D algorithms, their significance cannot be underestimated in glaucoma detection.

Despite yielding robust results, this study is not devoid of limitations. For instance, our analysis indicated that the 3D-ResNet18 and 3D-DenseNet121 models exhibited severe tendencies towards overfitting when applied to the ONH-OCT dataset. These findings suggest that the inherent complexity of the model may not be ideally suited for this dataset, featuring a resolution of 64 × 64 × 128. Therefore, the study could have benefited from a higher resolution and larger ONH-OCT dataset. These outcomes reiterate the importance of model selection in achieving optimal performance.

Another limitation is the scarcity of the large OCT volume dataset and pre-trained state-of-the-art (SOTA) 3D-CNN models. While it is plausible that a 3D model pre-trained on a comprehensive dataset could significantly enhance the model's accuracy, further research is required. Therefore, in future studies, we intend to explore additional models and datasets as they become available. Additionally, we also plan to pre-train a 3D-CNN model from scratch by curating a substantial corpus of medical and other volumetric image datasets to improve models’ performance in smaller glaucoma-specific volumetric datasets. These will provide more comprehensive insights into the comparative performance between 2D and 3D-CNN counterparts. Nevertheless, based on the publicly available glaucoma datasets and the ML resources readily available at our disposal, it is evident that 2D-CNN algorithms consistently deliver better results in glaucoma detection compared to the 3D-CNN algorithms and the weight transfer method utilized in this study for these datasets.

In conclusion, our investigation provides a thorough understanding of the strengths of 2D and 3D-CNN architectures in OCT-based glaucoma detection. Despite the loss of volumetric information in each 2D B-scan derived from the OCT volume; the 2D-CNN model still provides robust results by aggregating the prediction of all B-scans. The higher accuracy of 2D-CNN models underscores their potential to drive advancements in glaucoma diagnosis and management, especially considering the scarcity of pre-trained 3D CNN models trained on adequately large datasets and the constraints posed by the smaller sizes of glaucoma-specific datasets. It is also essential to emphasize that while the 2D approach yields excellent results, the selections of 2D and 3D ML algorithms are inherently problem-specific, demanding continuous efforts for ongoing progress. Therefore, by shedding light on the comparative performance of select 2D and 3D-CNN models on these smaller glaucoma datasets, our research aids in refining diagnostic tools in ophthalmology by highlighting the robust performance of 2D-CNN models and providing probable future direction to improve the performance of 3D-CNN models, thus, making a significant stride towards improving glaucoma detection techniques.

## Materials and methods

### Macular OCT data

For the macular-OCT dataset, we utilized the UKB dataset^30^, an ongoing project comprising health records of over half a million individuals aged between 40 and 70 years. Within this extensive dataset, a subset of spectral domain macular-OCT images was released for approximately 87,000 participants, captured between 2009 to 2013 using the TOPCON 3D OCT 1000 Mk2 device. Each OCT volume consists of 128 B-scans, of which each represents a 512 × 650 pixel grayscale image. As the B-scan index advances from 0 to 127, the OCT B-scans progressively shift from the superior region down to the inferior region. Our access and use of the UKB data were approved under application #23424 in accordance with their Access Procedures and Ethics regulations. We obtained fully de-identified data. Informed consent was obtained from the participants for their participation in the study by the UKB Committee upon recruitment. The study protocol was approved by The North West Multi-centre Research Ethics Committee.

**Figure 2:**
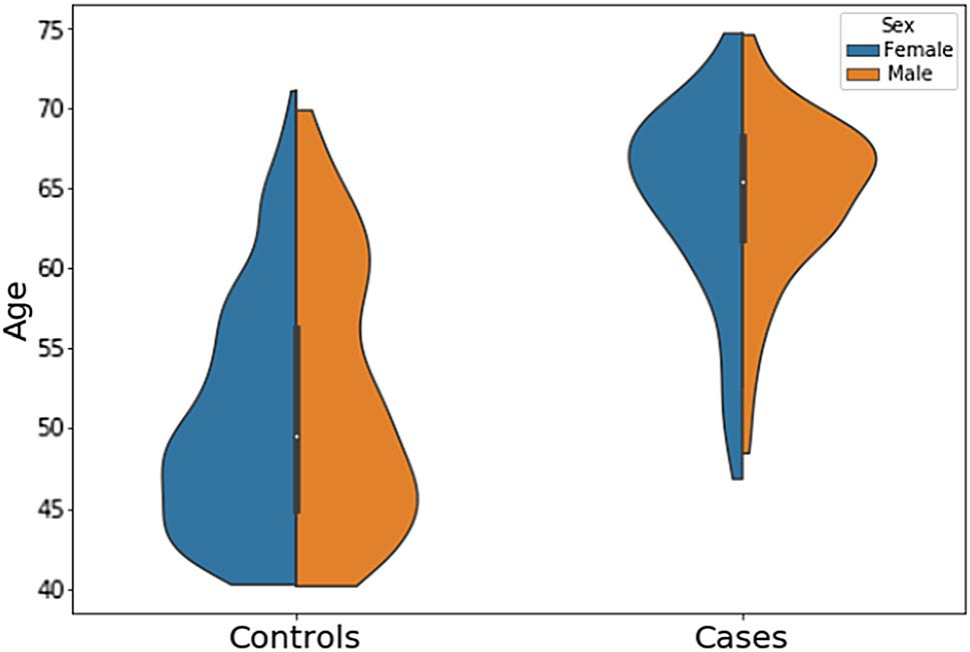
Age and sex distribution of the UKB study sample. The figure demonstrates the age and sex distribution present within the glaucoma (cases) and non-glaucoma (controls) individual in the UKB dataset.

In our cohort selection, we extracted participants with OCT scans from the UKB dataset consisting of both glaucoma cases and healthy individuals. Glaucoma cases were identified by the ICD-10 code H40.1, indicative of primary open-angle glaucoma (POAG). The healthy participants in our study had not reported any glaucoma or other eye-related conditions. Building upon previous research, we further refined our selection of healthy participants by excluding those who reported secondary health issues, such as high blood pressure or obesity, and those whose visual acuity was reported worse than 20/30 on the logMAR chart. After these exclusions, our dataset comprised of 255 individuals with glaucoma (~ 448 eyes) and 2,812 healthy participants (~ 5,619 eyes). For our research, we randomly chose 765 healthy individuals (about three times the number of cases encompassing ~ 1,530 eyes) from the healthy group and incorporated all 255 participants diagnosed with POAG. Figure 2 displays the age and sex distribution of the study sample.

### Optic nerve head OCT data

For the ONH-OCT, we utilized the publicly available OCT image dataset provided by Maetschke et al.^22^. This ONH-OCT dataset contains a collection of 1110 OCT volumes derived from 624 patients, imaged using a Cirrus SD-OCT Scanner (Zeiss, Dublin, CA, USA), focusing on the ONH region. Each publicly available OCT volume corresponds to a down-sampled version of 64 × 64 × 128 voxels, originating from the 200 × 200 × 1024 voxel dimensions. The dataset includes 263 scans representing healthy individuals and 847 cases were attributed to primary open-angle glaucoma (POAG) based on the provided labels. Demographic information, such as gender and race distribution, as well as mean values and standard deviations for patient age, intraocular pressure, and visual field test results, have previously been reported^22^.

### Train, validation, and test splits

We employed a tenfold cross-validation (CV) strategy to train and assess the performance of 2D and 3D-CNN algorithms on the macular-OCT and ONH-OCT datasets. Within each fold, we used stratified splitting to ensure a balanced representation of healthy and glaucomatous individuals and partitioned the dataset into 80% for training, 10% for validation, and 10% for testing. Special precautions were taken to ensure that data from eye images of a single individual were allocated to the same data split.

### Data preprocessing

Before model training, each B-scan and OCT volume underwent a series of preprocessing steps. Initially, images were cropped and resized to 224 × 224 pixels to align with the specifications of models pretrained on the ImageNet1k dataset. This was followed by standardizing the images to ensure their intensity ranges matched those of the images used for pretraining. To enhance the models' ability to generalize, data augmentation techniques were employed, such as random horizontal and vertical flips, rotations, and translations. While the data augmentation and preprocessing pipeline was similar for both 2D and 3D models, special attention was given to ensure that all slices within a given volume underwent identical augmentations during the training of 3D models.

### 2D Models

The primary DL architecture chosen for our 2D model is ResNet18, a convolutional neural network consisting of 18 deep layers. ResNet18 is part of the ResNet (Residual Network) family, known for its utilization of residual blocks^27,28^ to address the vanishing gradient problem common in deep models, thereby facilitating the training of very deep networks. The selection of ResNet18 is driven by its relative simplicity and efficiency in model training and previously demonstrated robust performance in disease detection^34,35^. This choice enhances its resistance to overfitting when working with smaller datasets.

**Figure 3:**
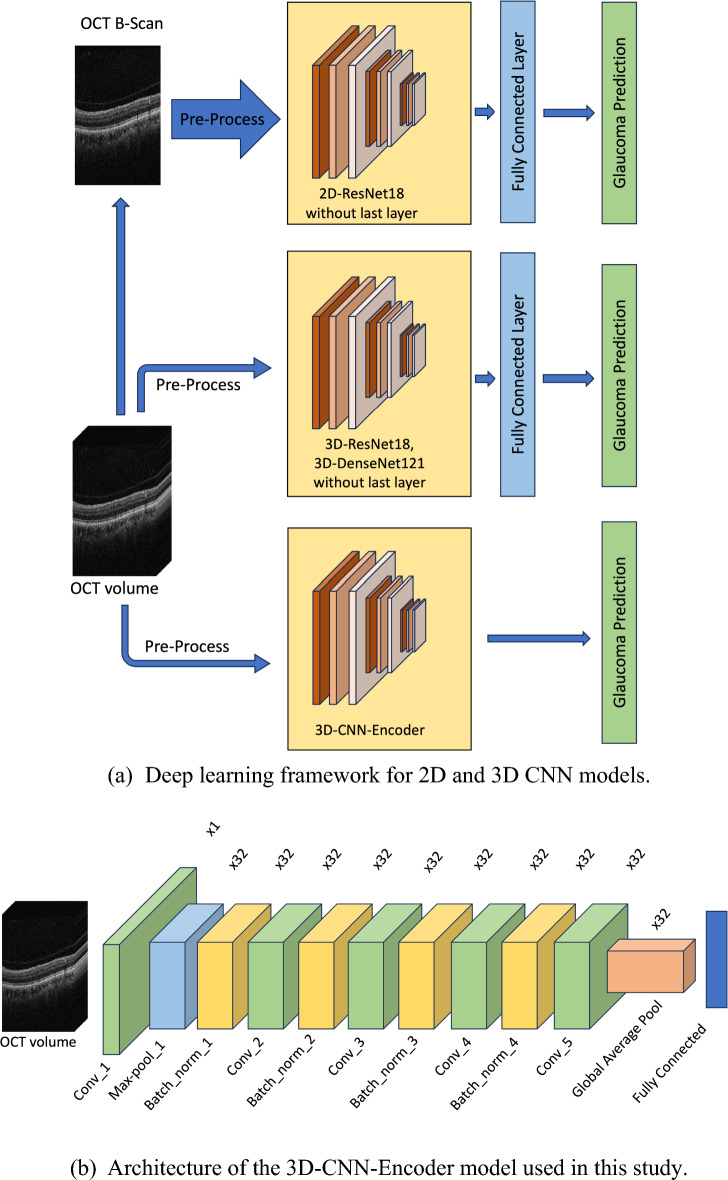
Deep learning framework utilized with the 2D and 3D CNN models. This figure illustrates the deep learning (DL) framework utilized with the 2D and 3D CNN models. Subfigure (**a**) displays the DL framework employed in this study, encompassing a data preprocessing pipeline, responsible for both data preprocessing and augmentation before the images are input into the model. Following this, a DL backbone (specifically, ResNet18 sourced from PyTorch’s torchvision library) is used for feature extraction, culminating in a final fully connected layer dedicated to glaucoma prediction. In contrast, subfigure (**b**) showcases the 3D-CNN-Encoder model, which is built from scratch and structured around five convolutional layers and a fully connected layer for the final prediction. *DL* deep learning, *CNN* convolutional neural network, *OCT* optical coherence tomography.

Our model begins with training on all B-scans (i.e., 128 for macular-OCT and 64 for ONH-OCT) collectively to generate per-B-scan glaucoma predictions. To achieve this, we consolidate each volume to match the corresponding number of B-scans and labeled each B-scan accordingly based on the labels of the OCT volume. As a result, after model training, the model can provide glaucoma predictions on a per-B-scan basis, rather than at the volume level. Therefore, these predictions are then aggregated using XGBoost^36^ to obtain final glaucoma prediction. We implemented the DL model using the PyTorch framework in Python and utilized a pre-trained ResNet18 model trained on the ImageNet1K dataset as the foundation for our architecture. The DL framework used in this work is depicted in Fig. 3.

During the fine-tuning process, we adopted a batch size of 64 and employed the Adam optimizer with an initial learning rate of 0.001 and an epsilon value of 0.1. The model underwent 50 epochs of training, with the best-performing model being determined based on the lowest loss observed in the training phase. For loss calculation, we employed the binary cross-entropy (BCE) loss function.

### XGBoost

To aggregate the results from all available OCT B-scan predictions obtained from the 2D model, XGBoost was trained and validated with a split between training and validation data. The hyperparameters for the macular-OCT-based XGBoost model were set as the following learning rate 0.01, maximum depth 3, subsample 0.7, alpha 0.1, and lambda 0.5. Similarly, the hyperparameters for the ONH-OCT-based XGBoost model were learning rate 0.01, maximum depth 3, subsample 0.7, colsample_bylevel 0.2, alpha 0.1, and lambda 0.5. Each XGBoost model was trained for 2000 epochs, and the best model was selected based on the lowest BCE loss.

### 3D-models

In this study, we explored two distinct 3D architectures: the 3D-ResNet18, 3D-DenseNet121^29^, and the 3D-CNN-Encoder. The 3D-ResNet18 and 3D-DenseNet121 are three-dimensional adaptations of their 2D counterparts, specifically designed to leverage the unique strengths of each architecture in volumetric contexts. In contrast to ResNet18, which utilizes residual connection, DenseNet121 employs a dense connectivity approach, directly linking each layer to every other, ensuring maximum information flow and excelling in detail-rich feature recognition due to its 121-layer depth^29^. To develop the 3D version, we replaced the 2D layers of the original model with 3D counterparts, maintaining the original model's essence but increasing the dimensionality. Transfer learning was also utilized; by employing dimensionality expansion, we adapted pretrained weights from the 2D CNN model to fit the 3D counterparts^24–26^. The weight transfer technique employed in this study mirrors a strategy elucidated by Ebrahimi et al.^24^, wherein they leveraged dimensionality expansion to iteratively replicate 2D CNN weights into their 3D equivalents. Xue et al.^25^ also utilized a similar weight transfer technique to enhance the performance of their 3D model. In both works, comprehensive training of all layers was undertaken. Given the inherent approximations associated with the adaptation of 2D weights to a 3D model, it is recommended to train all layers for optimal results. Moreover, Ebrahimi et al.^24^ and Xue et al.^25^ trained their models on 264 and 263 volumetric images, respectively, achieving robust results. This illustrates the weight transfer technique's success in training full models effectively on smaller datasets.

The 3D-CNN-Encoder architecture, depicted in Fig. 3b, is based on the foundational framework described in Maetschke et al.’s study^22^. This model consists of five convolutional layers, each incorporating a batch normalization layer, with the first layer including both a max pool and a batch normalization layer. Kernel sizes for these convolutional layers are set at 7 for the first, 5 for the second, and 3 for the others. The max pooling layer follows with a kernel size of 1 and a stride of 2. Each batch normalization is succeeded by a ReLU activation layer. The architecture concludes with a global average pooling layer feeding 32 features into the final fully connected layer, which is then used for predicting glaucoma.

During the fine-tuning process, we noticed that the hyperparameters for model training on both datasets converged to similar values. Consequently, we employed a batch size of 16 for both the 3D-ResNet18 and 3D-DenseNet121 models and a batch size of 64 for the 3D-CNN-Encoder model, all utilizing the Adam optimizer. The learning rates were set at 1e-5 for both the 3D-ResNet18 and 3D-DenseNet121 models and at 0.001 for the 3D-CNN-Encoder model, respectively. Each of these 3D models underwent 50 epochs of training, and we selected the best model based on achieving the lowest BCE loss.

### Performance metrics

The performance of the model was evaluated using a comprehensive set of metrics, including accuracy, sensitivity, specificity, and the area under the receiver operating characteristic curve (AUC-ROC). To generate ROC curves, we used the ‘roc_curve’ function from scikit-learn to plot the true positive rate (sensitivity) against the false positive rate (1-specificity) at various threshold settings. To determine the accuracy, sensitivity, and specificity of the model, we utilized the Youden Index^37^, which identifies the best balance between sensitivity and specificity for each model and each particular dataset. However, the Youden Index does not generalize effectively across different datasets and models. For this reason, it was utilized in this study primarily to support the results qualitatively, rather than serving as a definitive tool for validating the model's performance.

### Supplementary Information

Supplementary Information.

## Data Availability

The data used in this paper is publicly available except the UKB data which was obtained via contract using application ID #23424. Applications to access the data can be completed at: https://www.ukbiobank.ac.uk/enable-your-research/apply-for-access. Informed consent was obtained from the participants for their participation in the study by the UKB Committee upon recruitment. The study protocol was approved by The North West Multi-centre Research Ethics Committee.
