# Supplementary material for: Assessing the efficacy of 2D and 3D CNN algorithms in OCT-based glaucoma detection
Source: Sci Rep. 2024 May 23;14:11758. doi: 10.1038/s41598-024-62411-6 (PMC11116516; doi:10.1038/s41598-024-62411-6)
Supplement: Supplementary file 1 — Supplementary Information. [file 41598_2024_62411_MOESM1_ESM.docx]

**Web Resources**

The URLs for downloaded data

ONH-OCT (Public dataset, open access): <https://zenodo.org/record/1481223>

UK Biobank (Restricted access dataset, approved application #23424): <https://www.ukbiobank.ac.uk>
